# A partial least squares analysis of functional status, disability, and quality of life after surgical decompression for degenerative cervical myelopathy

**DOI:** 10.1038/s41598-020-72595-2

**Published:** 2020-09-30

**Authors:** Jetan H. Badhiwala, Omar Khan, Adam Wegner, Fan Jiang, Jamie R. F. Wilson, Benjamin R. Morgan, George M. Ibrahim, Jefferson R. Wilson, Michael G. Fehlings

**Affiliations:** 1 Division of Neurosurgery, Department of Surgery, University of Toronto, 149 College Street, 5th Floor, Toronto, ON M5T 1P5 Canada; 2 Spinal Program, Toronto Western Hospital, Krembil Neuroscience Center, University Health Network, 399 Bathurst Street, Suite 4W-449, Toronto, ON M5T 2S8 Canada; 3 Division of Neurosurgery, The Hospital for Sick Children, 555 University Avenue, Toronto, ON M5G 1X8 Canada; 4 Division of Neurosurgery, St. Michael’s Hospital, 30 Bond Street, Toronto, ON M5B 1W8 Canada

**Keywords:** Outcomes research, Spinal cord diseases

## Abstract

Previous studies aimed at identifying predictors of clinical outcomes following surgical decompression for degenerative cervical myelopathy (DCM) are limited by multicollinearity among predictors, whereby the high degree of correlation between covariates precludes detection of potentially significant findings. We apply partial least squares (PLS), a data-driven approach, to model multi-dimensional variance and dissociate patient phenotypes associated with functional, disability, and quality of life (QOL) outcomes in DCM. This was a post-hoc analysis of DCM patients enrolled in the prospective, multi-center AOSpine CSM-NA/CSM-I studies. Baseline clinical covariates evaluated as predictors included demographic (e.g., age, sex), clinical presentation (e.g., signs and symptoms), and treatment (e.g., surgical approach) characteristics. Outcomes evaluated included change in functional status (∆mJOA), disability (∆NDI), and QOL (∆SF-36) at 2 years. PLS was used to derive latent variables (LVs) relating specific clinical covariates with specific outcomes. Statistical significance was estimated using bootstrapping. Four hundred and seventy-eight patients met eligibility criteria. PLS identified 3 significant LVs. LV1 indicated an association between presentation with hand muscle atrophy, treatment by an approach other than laminectomy alone, and greater improvement in physical health-related QOL outcomes (e.g., SF-36 Physical Component Summary). LV2 suggested the presence of comorbidities (respiratory, rheumatologic, psychological) was associated with lesser improvements in functional status post-operatively (i.e., mJOA score). Finally, LV3 reflected an association between more severe myelopathy presenting with gait impairment and poorer mental health-related QOL outcomes (e.g., SF-36 Mental Component Summary). Using PLS, this analysis uncovered several novel insights pertaining to patients undergoing surgical decompression for DCM that warrant further investigation: (1) comorbid status and frailty heavily impact functional outcome; (2) presentation with hand muscle atrophy is associated with better physical QOL outcomes; and (3) more severe myelopathy with gait impairment is associated with poorer mental QOL outcomes.

## Introduction

Degenerative cervical myelopathy (DCM) is the leading cause of spinal cord injury and a major source of disability globally^1^. DCM refers to a clinical syndrome of neurological impairment related to chronic compression of the cervical spinal cord secondary to age-related osteoarthritic changes affecting the spinal column^2–4^. Over 70% of individuals over 65 years old have evidence of cervical degenerative changes, and approximately one-quarter of these people become clinically symptomatic from mechanical spinal cord compression^5^. As the global population continues to age^6^, optimizing the diagnosis and treatment of DCM are key public health priorities.

Operative intervention for DCM was traditionally performed with the goal of halting progression of neurological deficits^7^. However, more recent evidence^1, 8^ indicates that surgery actually improves function and quality-of-life (QOL); hence, surgical decompression has become standard of care for patients with moderate or severe symptomatic DCM. Nonetheless, there is substantial variability in the treatment outcomes of individual patients. There is hence a critical need to parse out this heterogeneity and identify factors associated with clinical improvement or worsening post-operatively. This would inform patient counselling and calibration of expectations, as well as facilitate selection of good surgical candidates.

An important limitation of previous studies is multicollinearity. This phenomenon refers to the existence of intrinsic correlations among predictor variables, which limits the ability to disentangle their independent contributions to outcome in a multi-variate regression^9^. Partial least squares (PLS) circumvents this limitation of traditional statistical models^10^. This mathematical approach is well suited to facilitate understanding of the interrelations among multi-dimensional data. PLS affords the opportunity to distill combinations of individual patient characteristics into common “phenotypes” that may be associated with outcomes. Further, as an advanced data-driven approach, PLS does not rely on any preconceived assumptions or hypotheses to test associations between predictors and outcomes^10, 11^. These features make PLS a potentially powerful tool in the analysis of complex relationships in epidemiological datasets; however, little work has been done in the application of PLS analysis to DCM specifically. Here, we apply PLS to dissociate phenotypes, defined by weighted combinations of demographic, disease, and treatment characteristics, associated with functional status, disability, and QOL outcomes in patients undergoing surgical decompression for DCM.

## Methods

### Data source & patient population

This was a post-hoc analysis of a dataset that combined the AOSpine CSM North America^1^ (CSM-NA; ClinicialTrials.gov NCT00285337) and AOSpine CSM International^12^ (CSM-I; ClinicalTrials.gov NCT00565734) studies. These were single-armed, prospective, multi-center cohort studies conducted at 26 global sites that aimed to evaluate the efficacy of surgical decompression in patients with DCM with regard to functional status, disability, and quality-of-life (QOL) outcomes. Approval for the study was obtained from the University of Toronto Research Ethics Board and the study was conducted in accordance with ethics guidelines. Patients were enrolled if they provided written informed consent and met the following eligibility criteria: (1) age 18 years or older; (2) symptomatic DCM with at least one clinical sign of myelopathy; (3) imaging evidence of cervical cord compression; and (4) no prior cervical spine surgery. All patients underwent surgical decompression of the cervical spine, with or without an instrumented fusion procedure.

### Clinical variables

Data pertaining to patient demographics (e.g., age, sex, BMI, education, comorbidities), clinical presentation (e.g., symptoms, signs, duration of myelopathy, causative pathology), and surgical treatment (e.g., approach, number of levels, operative duration) were obtained (Table 1).

**Table 1:** Predictor (X) and outcome (Y) variables included in analysis.

Predictors (X)	Outcomes (Y)
1. Age (years)	1. mJOA
2. Female sex (yes/no)	2. SF-36 Physical Component Summary
3. Married (yes/no)	3. SF-36 Mental Component Summary
4. Caucasian race (yes/no)	4. SF-36 Bodily Pain
5. Education > 12 years (yes/no)	5. SF-36 Mental Health
6 Weight (kg)	6. SF-36 Vitality
7. Height (m)	7. SF-36 General Health
8. BMI (kg/m2)	8. SF-36 Physical Functioning
9. Baseline mJOA score (0–17)	9. SF-36 Role Emotional
10. Neck pain on history (yes/no)	10. SF-36 Role Physical
11. Hand numbness on history (yes/no)	11. SF-36 Social Functioning
12. Hand clumsiness on history (yes/no)	12. NDI
13. Gait impairment on history (yes/no)	
14. Arm paresthesias on history (yes/no)	
15. Lhermitte's phenomenon on history (yes/no)	
16. Weakness on history (yes/no)	
17. Duration of DCM symptoms	
18. Motor deficits on examination (yes/no)	
19. Hand muscle atrophy on examination (yes/no)	
20. Hyperreflexia on examination (yes/no)	
21. Hoffman sign on examination (yes/no)	
22. Babinski sign on examination (yes/no)	
23. Lower limb spasticity on examination (yes/no)	
24. Unstable gait on examination (yes/no)	
25. Comorbidities (yes/no)	
26. Cardiovascular comorbidity (yes/no)	
27. Hypertension (yes/no)	
28. Respiratory comorbidity (yes/no)	
29. GastrointestinaI comorbidity (yes/no)	
30. End stage renal disease (yes/no)	
31. Diabetes mellitus (yes/no)	
32. Psychological comorbidity (yes/no)	
33. Rheumatologic comorbidity (yes/no)	
34. Neurological comorbidity (yes/no)	
35. Smoker (yes/no)	
36. Congenital stenosis (yes/no)	
37. Spondylosis (yes/no)	
38. Disc herniation (yes/no)	
39. OPLL (yes/no)	
40. Ligamentum hypertrophy (yes/no)	
41. Subluxation (yes/no)	
42. Upper cervical spine compression (C1–4) (yes/no)	
43. Anterior surgical approach (yes/no)	
44. Posterior surgical approach (yes/no)	
45. Combined (anterior/posterior) surgical approach (yes/no)	
46. Laminectomy alone (yes/no)	
47. Laminectomy plus fusion (yes/no)	
48. Laminoplasty (yes/no)	
49. Operative duration (min)	
50. Number of operative levels	

### Outcomes

Functional status was assessed by the mJOA scale^13, 14^. Quality of life was evaluated by the Short Form-36 (SF-36)^15^ and disability by the Neck Disability Index (NDI)^16, 17^. These are both patient self-reported measures, the SF-36 being a generic health-related quality of life instrument, and the NDI being specific to neck conditions. The outcomes of interest were change in mJOA, SF-36 (all eight domains and two summary scales), and NDI from baseline to 2-year postoperative follow-up (Table 1).

### Partial least squares analysis

We applied a PLS analysis to better understand patient phenotypes associated with outcomes following surgical decompression for DCM. A PLS analysis has the distinct advantages of decomposing the correlation between a set of variables and extracting patterns of variable contributions to the overall relationship with outcome. Multi-dimensional associations between clinical covariates (predictors) and outcome measures were assessed using PLS for all subjects with complete baseline and 2-year follow-up data. Detailed methodology has been published previously^11, 18^.

#### Raw matrix construction

The data were separated into **X** (predictor) and **Y** (outcome) matrices. The **X** matrix included 478 subjects by 50 clinical covariates and the **Y** matrix included 478 subjects by 12 outcome variables. Data centering was performed, such that covariates with large absolute values do not dominate the analysis. To center continuous data, z-scores were generated from the sample distribution. Zero-centering was performed for binary and ordinal variables.

#### Correlation matrix construction

A heterogeneous correlation matrix, **R**, was calculated between **X** and **Y** data matrices using the hetcor function of the polycor package in R statistical software. Pearson product-moment correlations (between continuous variables), polyserial correlations (between continuous and ordinal variables), and polychoric correlations (between ordinal variables) were generated^19^.

#### PLS analysis

Single value decomposition (SVD) was performed on the heterogeneous correlation matrix to produce latent variables (LVs), or components, explaining the greatest amount of correlation between **X** and **Y** (the predictors and outcomes, respectively)^20, 21^ as follows:

$$\text{R = USV'}$$

The latent variable represents the relation between the outcome scores and predictor variables. The values of **U**, **S**, and **V** were derived through SVD. Each column (i) of the matrices **U** and **V** characterizes a single component. The vectors **V**(i) and **U**(i) represent the weights of the independent and dependent variables that make the greatest contribution to the latent variable, respectively. The diagonal of the matrix **S** contains the singular values. The effect size and amount of variance explained by each component may be derived by calculating the ratio of a single squared singular value to the sum of all squared singular values^20^.

### Statistical analysis

The significance of the latent variables was tested by bootstrapping with 5000 iterations, a process of sampling with replacement. For each bootstrap, as previously reported, the data were rotated by a Procrustes rotation to align the first three vectors of **U** and **V** to the SVD of the original non-bootstrapped data^18^. The first three vectors were chosen because they explained the majority of the variance in the original SVD. The resampling distribution was used to derive standard errors and 95% confidence intervals for the contributions of each variable to the component. A bootstrap ratio, the ratio of each element in **U** and **V** to its bootstrap-estimated standard error (similar to a z score), was calculated to estimate the statistical reliability of each variable weighted by its contribution to the overall latent variable^11, 18, 20^. Significance was determined using |z|> 3.29, corresponding to p < 0.001.

Plots were generated to depict contributions of the **X** and **Y** variables to the first 3 latent variables explaining the greatest amount of variance from the PLS analysis. Each plot graphs the bootstrap-estimated 95% confidence intervals of significant **X** and **Y** variables. SVD, bootstrapping, and plot generation were conducted using MATLAB software (Mathworks Inc., Natick, MA, USA).

## Results

A total of 478 patients met eligibility criteria. Baseline clinical and surgical characteristics of the study cohort are presented in Table 2 and outcomes are presented in Table 3. Thirty-seven percent of the patient cohort was female, and the mean age was 56.4 ± 11.7 years (mean ± standard deviation). The signs and symptoms of the patients were varied, but most patients experienced hand numbness (88.5%) and weakness (86.6%). An anterior surgical approach was the choice of treatment in 62.3% of cases, while three possible posterior surgical approaches were employed: laminectomy alone, laminectomy with fusion, and laminoplasty. Generally, the functional status of patients improved after surgery, with the mean mJOA rising from 12.7 at baseline to 15.2 post-operatively.

**Table 2:** Baseline clinical and surgical characteristics of the patient cohort.

Variable	Mean ± standard deviation or frequency (proportion)
Age (years)	56.4 ± 11.7
Female sex	177 (37.0)
BMI (kg/m^2^)	27.4 ± 5.3
Married	352 (73.6)
Caucasian race	383 (80.1)
Education > 12 years	187 (39.1)
Duration of DCM symptoms (months)	28.3 ± 39.8
**Features on history**	
Neck pain	134 (28.0)
Hand numbness	423 (88.5)
Hands clumsiness	354 (74.1)
Gait impairment	364 (76.2)
Arm paresthesias	282 (59.0)
LHermitte’s phenomenon	129 (27.0)
Weakness	414 (86.6)
**Physical exam findings**	
Motor deficits	289 (60.5)
Hand muscle atrophy	176 (36.8)
Hyperreflexia	385 (80.5)
Positive Hoffman sign	290 (60.7)
Positive Babinski sign	171 (35.8)
Lower limb spasticity	219 (45.8)
Unstable gait	281 (58.8)
**Comorbidities**	183 (38.3)
**Cardiovascular comorbidities**	217 (45.4)
Hypertension	135 (28.2)
Respiratory comorbidities	50 (10.5)
Gastrointestinal comorbidities	71 (14.9)
End-stage renal disease	12 (2.5)
Diabetes mellitus	42 (8.8)
Psychiatric comorbidities	64 (13.4)
Rheumatologic comorbidities	22 (4.6)
Neurological comorbidities	28 (5.9)
Smoker	121 (25.3)
**Surgical approaches**	
Anterior	298 (62.3)
Posterior	194 (40.6)
Laminectomy only	15 (3.1)
Laminectomy with fusion	143 (30.0)
Laminoplasty	43 (9.0)
Combined (anterior/posterior)	14 (2.9)
Baseline mJOA	12.7 ± 2.7
**Pathology**	
Congenital stenosis	43 (9.0)
Spondylosis	392 (82.0)
Disc	337 (70.5)
OPLL	112 (23.4)
Ligamentum hypertrophy	131 (27.4)
Subluxation	27 (5.6)
Operative duration (minutes)	186.2 ± 80.0
Number of operated levels	2.7 ± 1.3

Continuous variables are represented using mean ± standard deviation while categorical variables are represented by their frequency and proportion (in percent) in the 478 patient cohort.

**Table 3:** Summary of 2-year outcomes of the patient cohort, represented using mean ± standard deviation. Δ denotes the change at 2-years relative to baseline.

Outcome at 2 years	Mean ± standard deviation
**ΔmJOA**	2.5 ± 2.7
**ΔSF36 scores**	
Physical component summary	5.8 ± 10.7
Mental component summary	5.3 ± 13.2
Bodily pain	6.7 ± 12.2
Mental health	6.1 ± 12.4
Vitality	5.0 ± 12.2
General health	1.9 ± 10.8
Physical functioning	6.4 ± 11.8
Role emotional	5.8 ± 15.6
Role physical	8.1 ± 13.7
Social functioning	6.5 ± 14.1
**ΔNDI**	− 12.2 ± 19.0

**Figure 1:**
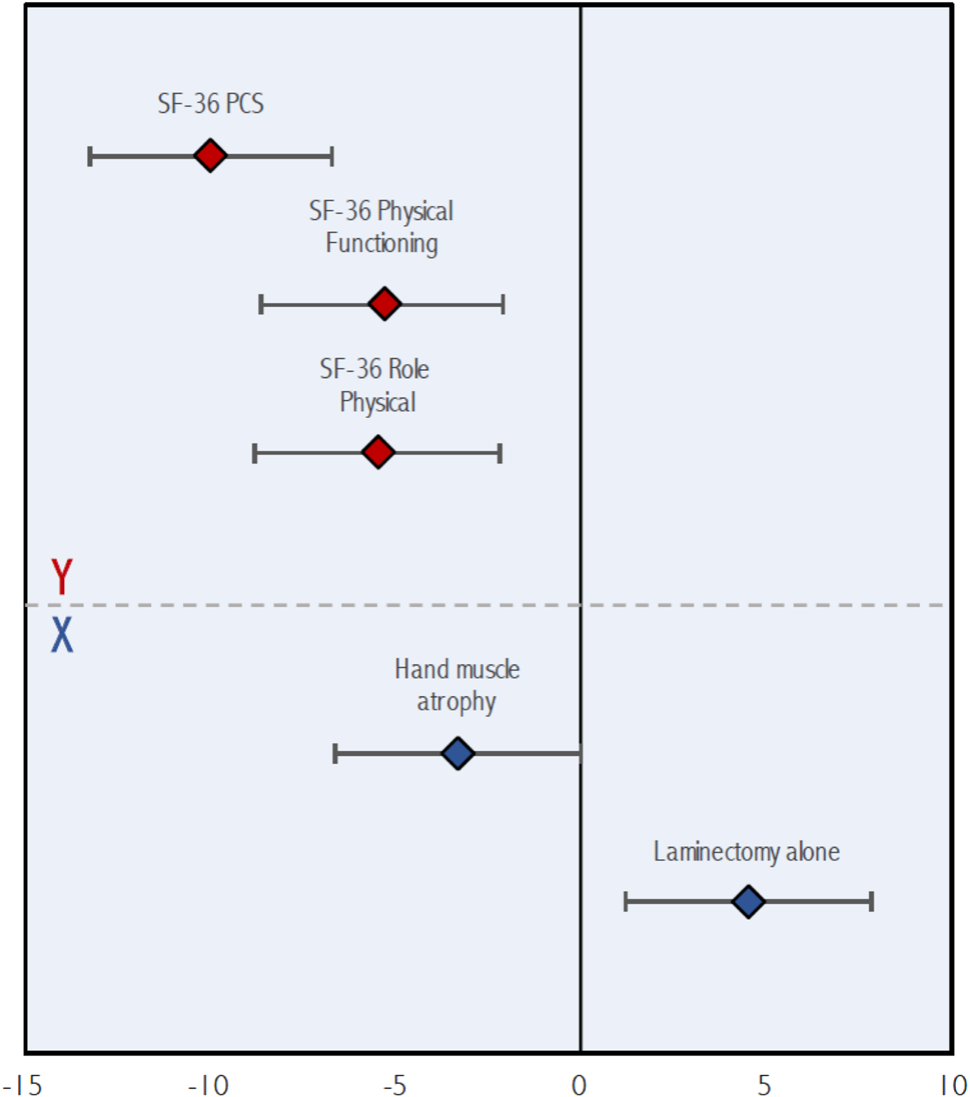
Significant contributions of the predictor (**X**) and outcome (**Y**) variables to the first latent variable. Mean values of outcome variables (bootstrap ratios) are shown with red diamonds while those for predictor variables are shown with blue diamonds. Error bars denote 95% confidence intervals.

**Figure 2:**
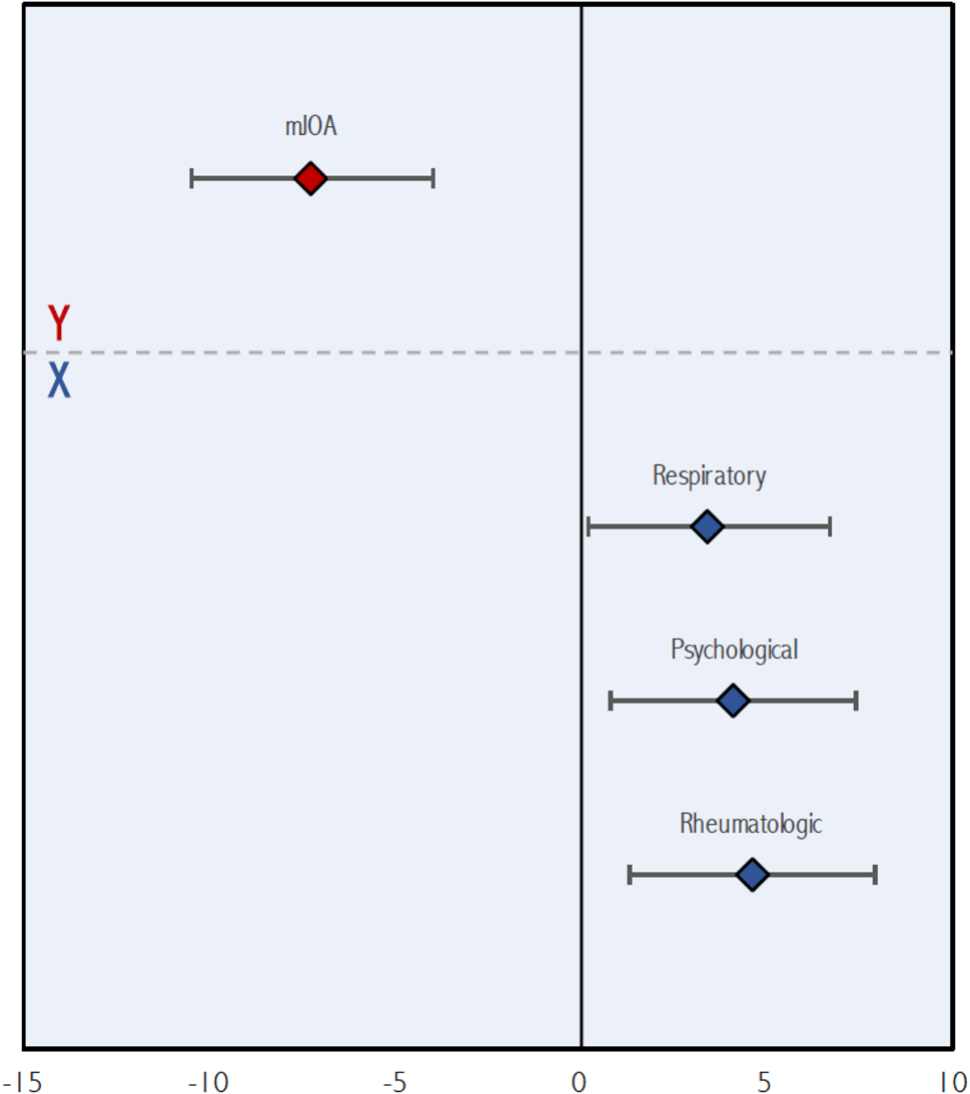
Significant contributions of the predictor (**X**) and outcome (**Y**) variables to the second latent variable. Mean values of outcome variables (bootstrap ratios) are shown with red diamonds while those for predictor variables are shown with blue diamonds. Error bars denote 95% confidence intervals.

**Figure 3:**
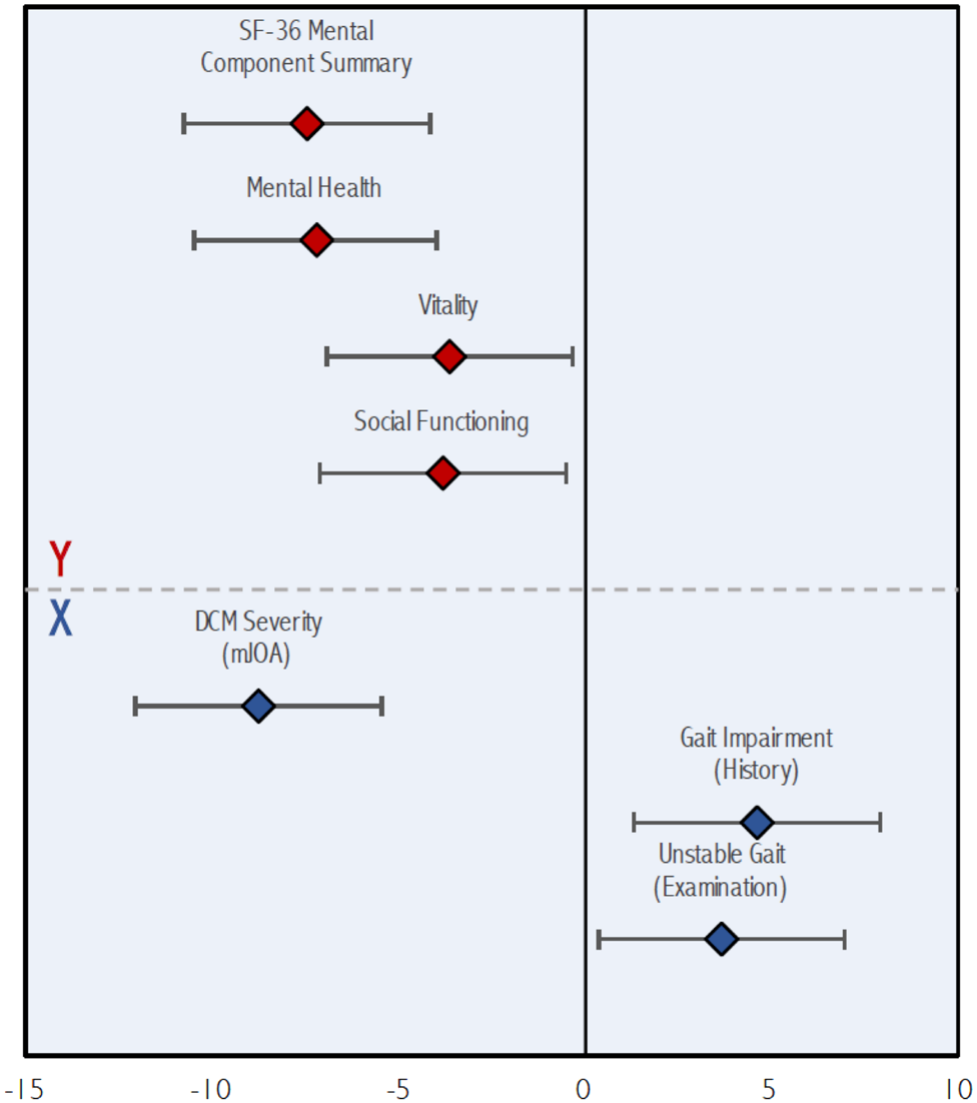
Significant contributions of the predictor (**X**) and outcome (**Y**) variables to the third latent variable. Mean values of outcome variables (bootstrap ratios) are shown with red diamonds while those for predictor variables are shown with blue diamonds. Error bars denote 95% confidence intervals.

Three significant latent variables were identified by PLS. Bootstrap ratios for predictors and outcomes for each latent variable are presented in Table 4. The first latent variable (Fig. 1) demonstrated that the phenotype of patient with intrinsic hand muscle atrophy (z = − 3.33, p < 0.001) treated by a surgical approach other than laminectomy alone (z = 4.51, p < 0.001) had greater improvement at 2 years in physical health-related QOL, including the Physical Component Summary (z = − 9.98, p < 0.001), Physical Functioning (z = − 5.35, p < 0.001), and Role Physical (z = − 5.51, p < 0.001) domains of the SF-36. Figure 2 plots the second latent variable. This revealed that patients with respiratory (z = 3.43, p < 0.001), rheumatologic (z = 4.59, p < 0.001), and psychological (z = 4.09, p < 0.001) comorbidities had poorer improvement in functional status post-operatively, as measured by the mJOA scale (z = 7.26, p < 0.001). Finally, in the third latent variable (Fig. 3), a phenotype of a patient with more severe myelopathy at presentation (lower mJOA) (z = − 8.80, p < 0.001) presenting with a history of gait impairment (z = 4.59, p < 0.001) and exam findings of a broad-based, unstable gait (z = 3.66, p < 0.001) was associated with poorer mental health-related QOL, as evaluated by change in the Mental Component Summary (z = − 7.44, p < 0.001), Mental Health (z = − 7.25, p < 0.001), Vitality (z = − 3.66, p < 0.001), and Social Functioning (z = − 3.81, p < 0.001) domains of the SF-36 at 2 years.

**Table 4:** Bootstrap ratios for predictor and outcome variables for each latent variable.

Variable	LV 1	LV 2	LV 3
**Predictors (X)**			
Age (years)	1.40	2.42	0.64
Female sex (yes/no)	0.02	0.41	1.98
Married (yes/no)	0.38	− 0.95	0.78
Caucasian race (yes/no)	0.25	0.85	0.49
Education > 12 years (yes/no)	1.53	0.19	1.07
Weight (kg)	1.58	0.39	1.41
Height (m)	1.66	− 0.02	0.32
BMI (kg/m^2^)	0.68	0.47	1.57
Baseline mJOA score (0–17)	0.95	0.98	− **8.80**
Neck pain on history (yes/no)	− 2.37	0.05	− 0.66
Hand numbness on history (yes/no)	1.06	− 1.95	2.17
Hand clumsiness on history (yes/no)	0.27	0.40	2.75
Gait impairment on history (yes/no)	− 2.27	− 0.74	**4.59**
Arm paresthesias on history (yes/no)	1.06	0.35	2.93
Lhermitte's phenomenon on history (yes/no)	− 0.95	1.66	0.19
Weakness on history (yes/no)	− 1.31	− 0.48	2.93
Duration of DCM symptoms	0.80	1.16	− 0.72
Motor deficits on examination (yes/no)	− 0.41	− 0.72	1.40
Hand muscle atrophy on examination (yes/no)	− **3.33**	− 0.44	1.09
Hyperreflexia on examination (yes/no)	− 2.00	− 0.49	− 0.08
Hoffman sign on examination (yes/no)	− 2.11	0.18	0.57
Babinski sign on examination (yes/no)	− 1.33	1.41	1.41
Lower limb spasticity on examination (yes/no)	− 0.66	0.21	0.47
Unstable gait on examination (yes/no)	0.00	0.42	**3.66**
Comorbidities (yes/no)	− 0.55	− 3.19	− 2.35
Cardiovascular comorbidity (yes/no)	0.97	0.46	1.56
Hypertension (yes/no)	0.90	− 1.57	− 0.06
Respiratory comorbidity (yes/no)	2.59	**3.43**	1.55
Gastrointestinal comorbidity (yes/no)	1.17	0.11	1.62
End stage renal disease (yes/no)	− 2.01	− 1.16	1.95
Diabetes mellitus (yes/no)	1.79	− 0.03	0.13
Psychological comorbidity (yes/no)	1.00	**4.09**	1.52
Rheumatologic comorbidity (yes/no)	− 1.91	**4.59**	− 1.26
Neurological comorbidity (yes/no)	− 0.96	0.13	0.49
Smoker (yes/no)	0.24	2.72	0.26
Congenital stenosis (yes/no)	0.19	1.21	0.59
Spondylosis (yes/no)	1.61	0.80	0.45
Disc herniation (yes/no)	− 0.63	− 2.02	0.65
OPLL (yes/no)	0.88	− 0.70	− 2.62
Ligamentum hypertrophy (yes/no)	1.60	− 0.43	0.22
Subluxation (yes/no)	− 1.44	0.25	0.01
Upper cervical spine compression (C1–4) (yes/no)	− 0.55	2.14	− 0.03
Anterior surgical approach (yes/no)	− 0.73	− 0.51	0.10
Posterior surgical approach (yes/no)	− 0.62	0.93	− 0.53
Combined (anterior/posterior) surgical approach (yes/no)	− 2.23	0.45	− 0.70
Laminectomy alone (yes/no)	**4.51**	0.25	0.02
Laminectomy plus fusion (yes/no)	− 1.93	2.50	− 0.58
Laminoplasty (yes/no)	− 0.88	− 0.91	− 0.21
Operative duration (min)	− 2.09	0.87	0.67
Number of operative levels	− 0.44	2.85	0.60
**Outcomes**			
mJOA	− 0.54	**7.26**	− 1.06
SF-36 physical component summary	− **9.98**	− 0.62	1.26
SF-36 mental component summary	1.65	− 0.09	− **7.44**
SF-36 bodily pain	− 1.44	− 1.42	− 2.31
SF-36 mental health	2.66	0.42	− **7.25**
SF-36 Vitality	− 0.46	− 0.74	− **3.66**
SF-36 general health	− 1.42	0.32	− 0.93
SF-36 physical functioning	− **5.35**	1.92	0.97
SF-36 role emotional	− 1.76	− 0.40	− 1.90
SF-36 role physical	− **5.51**	− 1.34	− 0.51
SF-36 social functioning	− 1.81	0.99	− **3.81**
NDI	0.67	− 0.24	2.83

## Discussion

A critical strength of PLS is its ability to overcome problems relating to multicollinearity; therefore, unlike traditional regression techniques, PLS does not require any preformed hypotheses or assumptions regarding the importance of particular variables^10, 11^. As a purely data-driven method, PLS hence has the potential to unveil novel associations that may not be intuitive, hypothesized to exist, or readily apparent. Further, PLS is able to examine multiple outcome variables simultaneously and dissociate the unique associations of a set of predictor variables with each. Here, we have applied PLS to disentangle multi-dimensional associations between predictors and outcomes in patients undergoing surgical decompression for DCM. This analysis has revealed several novel findings warranting further exploration and investigation. Specifically, a PLS approach dissociated the interrelations between baseline variables and three distinct aspects of a patient’s outcome: (1) functional status; (2) physical QOL; and (3) mental QOL.

Older age has been associated with poorer functional outcomes in patients with DCM^22, 23^. However, older patients generally have greater medical comorbidities and poorer physiological reserves; and it is unclear from the current literature whether age in and of itself is predictive of outcomes, or whether this influence is a biproduct of the association of age with comorbid status^24, 25^. The findings of the current study, which controlled for a host of baseline variables and distilled multi-dimensional associations, would support the latter. Indeed, it is interesting that in our analysis, age was not a significant predictor, whereas the second latent variable found a phenotype of respiratory, psychological, and rheumatologic comorbidities was strongly associated with lesser gains in functional status, namely change in mJOA score, following surgical decompression. Recently, the concept of ‘frailty’ has gained traction, which refers to a multi-faceted loss of reserves (e.g., energy, physical ability, cognition, health) giving rise to vulnerability^26^. Frailty is perhaps best conceptualized as an assessment of one’s ‘physiological age’. Studies of patients undergoing spine surgery for various pathologies have found that frailty may be more important than chronological age in predicting clinical outcomes^27, 28^. Similarly, the findings of the second latent variable are perhaps most appropriately interpreted as increasing frailty may lead to poorer functional gains following surgery for DCM. An explanation may be that patients who are more frail are less able to translate neurological recovery into functional gains; in other words, in these patients, improvements in motor or sensory function may not necessarily translate into a meaningful change in functional abilities.

Previous studies have found that more severe DCM-related impairment and longer duration of symptoms are associated with poorer surgical outcomes; most of these studies have focused on mJOA score as the outcome^22, 29, 30^. Using PLS, the present paper was able to delve somewhat deeper and provide novel insights. From the current analysis, it appears that the severity of symptoms, but also the pattern of impairment, is important in prediction outcomes; and further, that there is a differential impact on physical versus mental QOL outcomes.

First, based on the third latent variable, patients with more severe myelopathy at baseline, as assessed by the mJOA scale, and in particular, with subjective and objective impairment of gait, may have poorer mental QOL outcomes. Multiple prior studies have identified the presence of gait impairment to be a negative prognostic factor^13, 30^; and further, there is data to suggest that gait is less likely to recover in patients with more severe myelopathy^31^. The presence of non-recovering gait deficits post-operatively could understandably impact one’s self-perceived quality of life, and the impact may be disproportionately borne by mental and emotional facets of QOL. The presence of significant unsteadiness and/or need for assistive ambulation devices, for example, could limit one’s ability to engage in social activities, both within and outside the home, adversely impacting one’s overall emotional well-being. Based on these findings, DCM patients presenting with significant gait impairment should be counselled on the poorer mental QOL outcomes in order to calibrate expectations; and moreover, it may be necessary to more closely monitor these patients for the development of depressive symptoms.

Second, from the first latent variable, presentation with hand muscle atrophy was associated with greater improvement in physical QOL following surgical decompression. Hand muscle atrophy in DCM is thought to be due to multi-segmental compression affecting the ventral nerve roots or anterior horn of the spinal cord^32–34^. In general, this is felt to be self-limited and respond favorably to intervention^32, 35^. Based on the current analysis, it appears that patients presenting with hand muscle atrophy may have significant impairments in physical QOL that improve substantially following surgical decompression. Interestingly, surgical approach was a significant variable within this latent variable, with approaches other than laminectomy alone being associated with greater improvements in physical QOL. This association may be explained by the fact that patients with hand muscle atrophy often present with multi-segmental compression of the ventral nerve roots warranting either an anterior or a posterior multi-level approach (e.g., laminoplasty, laminectomy with fusion), rather than a simple laminectomy alone^32–35^. Nonetheless, by the nature of PLS, it is not possible to determine the independent effects of hand muscle atrophy versus laminectomy alone within this latent variable. Rather, one may conclude that the presence of hand muscle atrophy went hand-in-hand with surgical approaches excluding laminectomy alone, and these together were associated with more favorable physical QOL outcomes. The corollary is that the use of laminectomy alone as a surgical approach was associated with lack of hand muscle atrophy as a presenting symptom, and these together were associated with poorer physical QOL outcomes.

A discussed previously, the key strength of this paper is the use of PLS to explain multi-dimensional variance. This approach has not been applied to examine associations between predictors and outcomes in DCM previously, and accordingly, has revealed several novel insights. Nonetheless, this paper does have important limitations. First, all patients included in this analysis were treated surgically, and accordingly, the associations unveiled here do not apply to the natural history of DCM; that is, patients who are treated non-operatively. Further, because PLS by nature is a data-driven approach, these findings are exploratory in nature and warrant further investigation and validation, ideally in a prospective fashion.

## Conclusions

Using a data-driven approach, we were able to disentangle multi-dimensional associations between predictors and outcomes in patients undergoing surgical decompression for DCM. This revealed several novel insights: (1) comorbid status and frailty adversely impact gains in functional status; (2) presentation with hand muscle atrophy as a symptom is associated with greater improvements in physical quality of life; and (3) more severe myelopathy with gait impairment is associated with poorer mental quality of life outcomes. These findings warrant further investigation.
